# Long-term secondary prevention of acute myocardial infarction (SEPAT) – guidelines adherence and outcome

**DOI:** 10.1186/s12872-016-0400-6

**Published:** 2016-11-17

**Authors:** Constantinos Ergatoudes, Erik Thunström, Annika Rosengren, Lena Björck, Kristina Bengtsson Boström, Kristin Falk, Michael Fu

**Affiliations:** 1 Department of Molecular and Clinical Medicine, Institute of Medicine, Skövde, Sweden; 2 Institute of Health and Care Sciences, Sahlgrenska Academy, University of Gothenburg, Skövde, Sweden; 3 R & D Centre Skaraborg Primary Care, Skövde, Sweden; 4 Department of Medicine, Section of Cardiology, Sahlgrenska University Hospital/Östra, 41651 Göteborg, Sweden

**Keywords:** Secondary prevention, Cardiovascular disease, Myocardial infarction, Long-term

## Abstract

**Background:**

A number of registry studies have reported suboptimal adherence to guidelines for cardiovascular prevention during the first year after acute myocardial infarction (AMI). However, only a few studies have addressed long-term secondary prevention after AMI. This study evaluates prevention guideline adherence and outcome of guideline-directed secondary prevention in patients surviving 2 years after AMI.

**Methods:**

Patients aged 18–85 years at the time of their index AMI were consecutively identified from hospital discharge records between July 2010 and December 2011 in Gothenburg, Sweden. All patients who agreed to participate in the study (16.2%) were invited for a structured interview, physical examinations and laboratory analysis 2 years after AMI. Guideline-directed secondary preventive goals were defined as optimally controlled blood pressure, serum cholesterol, glucose, regular physical activity, smoking cessation and pharmacological treatment.

**Results:**

The mean age of the study cohort (*n* = 200) at the index AMI was 63.0 ± 9.7 years, 79% were men. Only 3.5% of the cohort achieved all six guideline-directed secondary preventive goals 2 years after infarction. LDL < 1.8 mmol/L was achieved in 18.5% of the cohort, regular exercise in 45.5% and systolic blood pressure <140 mmHg in 57.0%. Anti-platelet therapy was used by 97% of the patients, beta-blockers by 83.0%, angiotensin-converting enzyme inhibitors/angiotensin receptor blockers by 76.5% and statins by 88.5%. During follow-up, non-fatal adverse cardiovascular events (cardiac hospitalization, recurrent acute coronary syndrome, angina pectoris, new percutaneous coronary intervention, new onset of atrial fibrillation, post-infarct heart failure, pacemaker implantation, stroke/transient ischemic attack (TIA), cardiac surgery and cardiac arrest) occurred in 47% of the cohort and readmission due to cardiac causes in 30%.

**Conclusions:**

Our data showed the failure of secondary prevention in our daily clinical practice and high rate of non-fatal adverse cardiovascular events 2 years after AMI.

## Background

The main purpose of secondary prevention after acute myocardial infarction (AMI) is to reduce recurrence, decrease morbidity and mortality and improve quality of life. To achieve these goals, available guidelines for cardiovascular prevention worldwide uniformly recommend lifestyle interventions: smoking cessation, increased physical activity, maintaining a healthy body mass index (BMI), optimal control of risk factors (blood pressure, cholesterol and glucose control) and optimal use of cardio protective drug therapies (aspirin, beta-blockers, angiotensin-converting enzyme (ACE) inhibitors/angiotensin II receptor blockers (ARBs), lipid-lowering drugs) [1–5].

During the past decades, several studies have repeatedly demonstrated suboptimal adherence to guidelines in patients after AMI. EUROASPIRE (European Action on Secondary and Primary Prevention by Intervention to Reduce Events) I-IV surveys showed that a large majority of coronary artery disease patients did not achieve the standard of secondary prevention stipulated by the guidelines (1–5). The prevalence of smoking, poor diet and physical inactivity remains high and most patients remain overweight or obese with a high prevalence of diabetes. Moreover, despite increasing use of medications, risk factor control remains suboptimal [6–8]. A recent Swedish study showed that risk factor control has improved slightly over time after AMI, but improvement was mainly seen in control of blood pressure, indicating substantial potential for improvement in other preventive goals [9, 10].

Persistent secondary prevention is warranted after AMI for long-term cardiovascular protection. However, available studies that have evaluated secondary prevention have mainly been done up to within 1 year post AMI. Moreover, most studies have been registry-based, where data are often restricted and less detailed. Consequently, knowledge about secondary prevention 2 years post-AMI is limited. The main objectives of the current study were therefore to evaluate adherence to secondary prevention guidelines and outcome 2 years after AMI.

## Methods

### Patients

We included consecutive men and women who had undergone AMI, were aged ≥18 years and <85 years at the time of their index event and who were still alive 2 years post-AMI. The patients were identified retrospectively from hospital discharge lists. All participants were living in the catchment area of Gothenburg and had been hospitalized for AMI at Sahlgrenska University Hospital Östra or Sahlgrenska University Hospital/Sahlgrenska between July 2010 and December 2011. The diagnosis of AMI was based on the following criteria: at least one value of troponin level >15 ng/L and at least one of the following: chest pain, newly discovered ECG changes (pathological ST/T-wave, left bundle branch block or a pathological Q-wave), regional wall motion abnormality in the left ventricle discovered by echocardiography or MRI or proven intracoronary thrombosis or stenosis by coronary angiography [11]. Patients unable to speak or understand Swedish were excluded.

### Structured interview

A personal interview was conducted by experienced nurses at a minimum of 2 years after the index AMI event. A detailed questionnaire containing pre-defined questions was used that included information on marital status, living situation, education, occupation, past illnesses, chest pain and shortness of breath, depression, medication, smoking, use of Swedish snus, alcohol use, stress and exercise. The questionnaire was completed under supervision of the research staff.

Higher education was defined as having a university degree. Employment was coded dichotomously (working or not working). Smoking was defined as never smoked; yes, regularly; yes, sometimes; no, quit smoking. Quit smoking was defined as trying to quit during the 2 weeks preceding the interview. Depression was defined as feeling depressed for more than 2 consecutive weeks during the past 12 months. Self-perceived stress was defined as permanent stress during the last 5 years [12] Self-reported physical activity during leisure time was categorized into four levels according to a modified version of Saltin-Grimby Physical Activity Level Scale (SGPALS) [13]: sedentary; moderate physical activity at least 2 h per week (without sweating); regular physical activity (1–2 times per week); vigorous physical activity for at least 3 times per week.

### Structured examinations

A variety of variables were measured, including height, weight, waist circumference, hip circumference, blood pressure and ECG. Body height was measured to the nearest ½ cm and weight to 0.1 kg (Tanita Corporation, Tokyo, Japan), with the subjects in light clothing and without shoes. BMI was calculated as weight (kg)/height (m) squared. Waist was defined as the area between the iliac crest and to the approximate area of the lowest rib. Hip circumference was measured as the widest circumference around the hips. Resting ECG was taken with a 12-lead ECG (Cardiolex, Solna, Sweden). Blood pressure was measured twice for each person after a 5-min rest with the person in a sitting position using the Omron HEM-907 IntelliSense professional digital blood pressure monitor (Omron Corporation, Omuro, Kyoto, Japan).

### Laboratory analyses

Fasting blood samples were collected for analysis of hemoglobin, low-density lipoprotein (LDL) cholesterol, high-density lipoprotein (HDL) cholesterol, total triglycerides, total serum cholesterol, ApoB/ApoA1 ratio, glycated hemoglobin HbA1c, blood glucose, potassium, sodium and creatinine. HbA1c was measured in all participants because HbA1C is widely accepted as one of diagnostic criteria for diabetes. As long as patients have HbA1c < 48 mmol/mol they have achieved goals regardless of diabetes or not. Serum and plasma were aliquoted and stored at a −80 °C until analysed. All laboratory analyses were done at the Department of Clinical Chemistry, Sahlgrenska University Hospital, Gothenburg, Sweden.

### Data from medical records

A retrospective review of medical records was conducted to collect data on demographic details, medical history, diagnostic test results (e.g., ECG, echocardiography and coronary angiogram), treatments and outcome data.

### Outcome measures

The primary outcome measure was the proportion of patients with AMI who achieved the stipulated secondary preventive guideline recommendations, categorized as six variables (Table 1). The secondary outcome measure was non-fatal adverse cardiovascular events 2 years after AMI.

**Table 1:** Secondary prevention guidelines after AMI

1	Optimally controlled blood pressure, defined as SBP <140 mmHg
2	Optimally controlled cholesterol levels, defined as LDL cholesterol <1.8 mmol/L
3	Optimally controlled glucose, defined as HbA1c <48 mmol/mol
4	Regular physical activity that caused sweating at least two times a week
5	Smoking cessation, defined as non-smoking at the time of the interview
6	Pharmacological treatment with ACE inhibitors or ARBs

*AMI* acute myocardial infarction, *SBP* systolic blood pressure, *LDL* low-density lipoprotein, *ACE* angiotensin-converting enzyme, *ARB* angiotensin II receptor blockers

The definitions for achieving a guideline standard are presented in Table 1 [3]. Non-fatal cardiovascular events were defined as all cardiovascular events that occurred after the index AMI, including recurrent acute coronary syndrome, angina pectoris, new onset of atrial fibrillation, post-MI heart failure, percutaneous coronary intervention (PCI), cardiac arrest, stroke/TIA and any readmission secondary to cardiac disease. Post-MI heart failure was defined as a newly developed clinical manifestation of heart failure based on heart failure symptoms in combination with either increased NT pro BNP >300 pg/ml or LVEF <40% after AMI. Angina pectoris was defined as ≥ class 2 based on the Canadian Cardiovascular Society Angina Grading Scale (CCS Angina Grading Scale) [14]. Cardiac readmissions were all readmissions due to any cardiac cause (according to The International Classification of Diseases ICD-10).

### Statistical analyses

Data are reported as frequency and percentage for categorical variables and mean with standard deviation for quantitative variables. Data were analysed using IBM SPSS Statistics 22.0 (IBM Corp., Chicago, ILL, USA).

### Ethics

This study complies with the Declaration of Helsinki [15] and the study protocol was approved by the Ethics Committee of the Medical Faculty of the University of Gothenburg. Written informed consent was obtained from each participant by the principal investigator. The research assistants signed the Case Report Form to confirm that informed consent was obtained.

## Results

### Study cohort

**Fig. 1:**
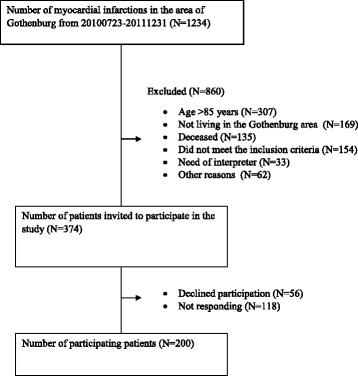
Study flow chart

In total, 1234 patients were hospitalized with an AMI in the area of Gothenburg between July 2010 and December 2011. Of those 1234 patients, 860 were excluded from the study because of various reasons (Fig. 1). After excluding all participants that either did not meet the inclusion criteria, or who could not be accessed, 374 patients were eligible. Of those, 56 patients did not sign the informed consent form or declined further participation and 118 did not respond to the invitation. Thus, the final sample included 200 patients (Fig. 1).

### Baseline characteristics at the time of the index AMI

At the time of the index AMI, 69% of the patients had ST-segment elevation myocardial infarction (STEMI), 31% a non–STEMI (NSTEMI). Mean heart rate was 75.6 (SD ±18.8) beats/min, mean systolic blood pressure (SBP) 146.1 (SD ±25.7) mmHg and mean diastolic blood pressure (DBP) 91.3 (SD ±15.0) mmHg. Almost everyone that was included (96%) underwent a percutaneous coronary intervention (PCI) or a coronary artery bypass grafting (CABG) in association with the index event.

### Demographic characteristics at interview

The mean age of the study cohort at follow-up (*n* = 200) was 65.5 (SD ±9.8) years and 79% were male (Table 2). Moreover, 79.3% were born in Sweden, 23.5% had a higher education and 34.4% were still actively working.

**Table 2:** Social and clinical characteristics of patients at the time of the interview 2 years after AMI

	Groups		
Variables	All *N* = 200	Men *N* = 158	Women *N* = 42
Age, years, mean ± SD	65.5 ± 9.8	64.7 ± 9.5	68.2 ± 10.2
Social factors			
Born in Sweden^a^ *n* = 199 (%)	158 (79.3)	123 (77.8)	35 (83.3)
Higher education^a^ *n* = 200 (%)	47 (23.5)	35 (22.2)	12 (28.6)
Currently working^a^ *n* = 199 (%)	72 (34.4)	62 (39.2)	10 (23.8)
Smoking^a^ *n* = 200			
Never (%)	62 (31.0)	45 (28.5)	17 (40.5)
Regularly (%)	13 (6.5)	13 (8.2)	0 (0.0)
Sometimes (%)	12 (6.0)	11 (7.0)	1 (2.4)
Quit (%)	113 (56.5)	89 (56.3)	24 (57.1)
Exercise^a^ *n* = 200			
Sedentary n (%)	28 (14)	18 (11.4)	10 (23.8)
Moderate activity n (%)	81 (40.5)	63 (39.9)	18 (42.9)
Regular activity n, (%)	50 (25.5)	41 (35.9)	9 (21.4)
Vigorous activity n, (%)	41 (20.5)	36 (22.8)	5 (11.9)
Cardiovascular diseases			
Atrial fibrillation^a^ *n* = 196 (%)	29 (14.8)	19 (12.3)	10 (24.4)
Known heart failure^a^ *n* = 193 (%)	16 (8.3)	10 (6.5)	6 (15.8)
Hypertension^a^ *n* = 194 (%)	123 (63.4)	93 (61.2)	30 (71.4)
Diabetes^a^ *n* = 197 (%)	43 (21.8)	37 (23.6)	6 (15)
Stroke/TIA^a^ *n* = 198 (%)	14 (7.2)	12 (7.7)	2 (4.9)
Hyperlipidemia^a^ *n* = 180 (%)	115 (63.9)	90 (63.4)	25 (65.8)
Previous MI *n* = 200 (%)	26 (13.0)	19 (12.0)	8 (19.0)
Previous PCI *n* = 200 (%)	23 (11.5)	18 (11.4)	5 (11.9)
Non-cardiovascular disease			
Cancer^a^ *n* = 200 (%)	31 (15.5)	22 (13.9)	9 (21.4)
COPD *n* = 193 (%)	39 (20.2)	29 (19.1)	10 (24.4)
OSA *n* = 162 (%)	48 (29.6)	37 (29.6)	11 (29.7)
Renal failure GFR < 60 *n* = 200 (%)	55 (27.5)	33 (20.1)	18 (42.9)
Renal failure GFR < 30 *n* = 200 (%)	4 (2)	4 (2.5)	0 (0.0)
Depressed for at least 2 consecutive weeks in the past 12 months^a^ *n* = 200 (%)	30 (15)	20 (12.7)	10 (23.8)

Data were presented as n (%)

*TIA* transient ischemic attack, *MI* myocardial infarction, *PCI* percutaneous coronary intervention, *COPD* chronic obstructive pulmonary disease, *OSA* obstructive sleep apnea

^a^Self-specified answers to questions in the SEPAT questionnaire

### Lifestyle characteristics and psychological conditions at interview

As shown in Table 2, a minority of the patients were still smokers (12.5%) regularly or occasionally; however, most of these patients had smoked at some time in their life (69%). Only one of seven patients reported a sedentary lifestyle 2 years after their index AMI, whereas 40.5% claimed to engage in moderate physical exercise without sweating, 25% did moderate exercise with sweating and 20.5% participated in regular physical exercise and training. Concerning psychological factors, 15% of the patients had been depressed during the past year and almost 10% had felt constantly stressed during the past 5 years. Feeling stressed and depressed were more common in those aged <65 years. Almost 20% of the patients <65 years of age had been constantly stressed for the past 5 years, whereas only 2% of those aged >65 years had felt stressed during the same period.

### Clinical characteristics based on interview, review of medical records and physical examinations

The two most common comorbidities were hypertension (63.4%) and hyperlipidemia (63.9%), followed by diabetes mellitus (21.8%) and atrial fibrillation (14.8%). Medical records showed that 22.5% of the patients were still smoking at the time of the index AMI, 13% had at least one previous myocardial infarction and 11.5% underwent PCI (Table 2).

At the physical examination, which was given during the interview, mean SBP was 137.5 mmHg and mean DBP 79.6 mmHg respectively (Table 3). Sinus rhythm constituted 93% with a mean heart rate at approximately 59.0 beats/minute. 17.5% of the study population had a BMI over 30 kg/m^2^

**Table 3:** Physical examination and laboratory analysis at the time of the interview 2 years after the index AMI event

	Groups		
	All *N* = 200	Men *N* = 158	Women *N* = 42
SBP (mmHg)	137.5 (18)	138.3 (18.3)	134.2 (16.4)
DBP (mmHg)	79.6 (10.3)	80.7 (10.0)	75.5 (10.5)
HR (beats/minute)	59.7 (10.7)	59.1 (10.8)	61.9 (10.2)
Weight (Kg)	84.7 (16.2)	86.9 (15.4)	76.4 (16.3)
BMI			
25 kg/m^2^ < BMI ≤30 kg/m^2^ (%)	47.0	50.6	35.7
> 30 kg/m^2^ (%)	25.5	20.9	40.5
Waist (cm)	100.2 (12.4)	101.4 (11.0)	95.7 (16.1)
Lab			
HbA1c (mmol/L)	40.5 (9.3)	39.8 (8.8)	43.0 (10.5)
Cholesterol (mmol/L)	4.1 (1.0)	4.1 (0.9)	4.5 (1.2)
LDL (mmol/L)	2.4 (0.9)	2.4 (0.8)	2.6 (1.1)
HDL (mmol/L)	1.4 (0.6)	1.4 (0.4)	1.6 (0.5)
ApoB/ApoA1 quote	0.6 (0.2)	0.6 (0.2)	0.5 (0.2)
Triglycerides (mmol/L)	1.3 (0.6)	1.3 (0.6)	1.3 (0.5)
P-glucose (mmol/L)	5.7 (1.3)	5.7 (1.3)	5.7 (1.3)
NTproBNP (pg/ml)	396.8 (750.7)	344.1 (682.3)	594.9 (949.6)
NTproBNP > 300 pg/ml (%)	31.5	25.3	44.2

Data were presented as n (%), otherwise in mean (SD). *SBP* systolic blood pressure, *DBP* diastolic blood pressure, *HR* heart rate, *BMI* body mass index, *LDL* low density lipoprotein, *HDL* high density lipoprotein

### Non-fatal cardiovascular events

During the 2-year study period non-fatal cardiovascular events occurred in 46.5% of the participants. Readmissions due to all causes were 50.5% and due to cardiac disease 30%. Among them, new AMIs constituted 8%, new PCIs 11.5%, atrial fibrillation 7.5%, TIA/stroke 6%, cardiac arrest 1%, post-infarction heart failure 19.5% and cardiac surgery 9.5% (Table 4). It is noteworthy to mention that the number of cardiac events may outnumber readmission rate since several events may occur during the same readmission. Our study was not powered to assess the causal relation between increased non-fatal cardiovascular events and suboptimal secondary prevention.

**Table 4:** Non-fatal cardiovascular events during the 2-year follow-up after the index AMI event

Readmissions, all cause, *n* (%)	101 (50.5)
Readmissions, cardiac, *n* (%)	60 (30.0)
Recurrent myocardial infarction, *n* (%)	16 (8.0)
Unstable angina, *n* (%)	8 (4.0)
PCI, *n* (%)	23 (11.5)
Cardiac surgery, *n* (%)^a^	19 (9.5)
Cardiac arrest, *n* (%)	2 (1.0)
Stroke/TIA, *n* (%)	9 (4.5)
Post-MI heart failure, *n* (%)	40 (20.0)
Post-MI atrial fibrillation, *n* (%)	15 (7.5)
Angina pectoris CCS ≥2, *n* (%)	34 (17.0)

*AMI* acute myocardial infarction, *PCI* percutaneous coronary intervention, *TIA* transient ischemic attack, *MI* myocardial infarction, *CCS* Canadian Cardiovascular Society

^a^cardiac surgery: all surgical procedure related to heart except percutaneous coronary intervention

### Achievements of guideline standards in secondary prevention

**Fig. 2:**
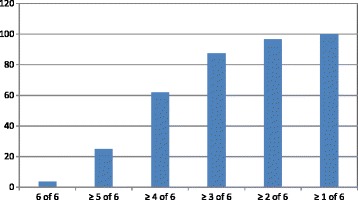
Number of achieved goals (%) of guideline standard secondary prevention two years post AMI

As shown in Table 5, the goal of SBP was achieved in 57% of the patients, 18.5% had LDL of <1.8 mmol/L, 87.5% did not smoke, 45.5% achieved the goal defined by regular exercise with sweating. The goal for HbA1c was achieved in almost 90% of the patients; however, most of these patients did not have diabetes. ACE inhibitor/ARB medication was administered to 75% of the patients. For other medications, 96% of the patients were treated with antiplatelet medicines, 83.0% with beta-blockers and 88.5% with statins. Only 3.5% of the whole study cohort achieved all six secondary preventive goals 2 years after AMI (Tables 5, 6, and Fig. 2).

**Table 5:** Development of a risk profile at the time of AMI and at the 2-year follow-up interview

	At AMI	At the 2-year follow-up interview
No smoking *n* (%)	155 (77.5)	175 (87.5)
LDL cholesterol <1.8 mmol/L *n* (%)	8 (4)	37 (18.5)
HbA1C <4.8 mmol/mol *n* (%)	NA	177 (88.5)
SBP <140 mmHg *n* (%)	152 (76)	114 (57)
Regular exercise training *n* (%)	NA	90 (45.5)

*NA* not available, *AMI* acute myocardial infarction**,** *LDL* low density lipoprotein, *SBP* systolic blood pressure

**Table 6:** Comparison of pharmacological treatments at the time of the index AMI event and at interview 2 years after AMI

	At AMI	At interview
Aspirin *n* = 200 (%)	199 (99.5)	183 (91.5)
Beta-blocker *n* = 200 (%)	187 (93.5)	166 (83.0)
ACE-inhibitor or Angiotensin receptor blocker *n* = 200 (%)	177 (88.5)	153 (76.5)
Statin *n* = 200 (%)	193 (96.5)	177 (88.5)
Clopidogrel/prasugrel *n* = 200 (%)	179 (89.5)	11 (5.5)

*AMI* acute myocardial infarction, *ACE* angiotensin-converting enzyme

### Development of risk profile 2 years after AMI

As shown in Table 5, there were more individuals that reached the goals of secondary prevention 2 years after the index AMI than at the time of AMI in terms of smoking, LDL reduction, blood pressure control and physical activity (Table 5). Moreover, there was a tendency towards increased BMI 2 years after the index AMI. The proportion of patients with BMI >30 kg/m^2^ increased from 17.5% at the time of the index AMI to 25.5% 2 years later.

## Discussion

In the present study the six secondary preventive goals were achieved by only 3.5% of the patients 2 years after the AMI event. Adherence to cardio-protective medications, however, was generally good. Still, non-fatal cardiovascular events occurred in 46.5% of the cohort and cardiac readmissions in 30% at the 2-year follow-up.

Our study is an extension of the EUROASPIRE survey and the Secondary Prevention after Heart Intensive Care Admission (SEPHIA) Registry in Sweden. EUROASPIRE IV was a cross-sectional study carried out in 24 European countries. Patients included in that study were those with either acute coronary syndrome or elective revascularization in the form of balloon angioplasty or coronary artery surgery. The starting date for identification of an event was ≥ 6 months and < 3 years before the expected date of the study interview [8]. The SEPHIA Registry is an on-going Swedish quality registry of AMI patients that focuses on four preventive goals (smoking cessation, optimal control of blood pressure, cholesterol and glucose) and data on the patients are collected during the first year after an AMI. Patients older than 75 years of age are not included in the Registry. Patients are invited to be interviewed by either a registered nurse or physician during an outpatient visit or via a telephone call on two occasions: 4–10 weeks (mean 56 days) and 12–14 months (mean 399 days) after myocardial infarction [9].

Our findings extended available observations by showing divergent development of risk factors from short-term to long-term time frame after AMI. Firstly, our study confirmed some observations from previous short-term studies. For instance, regular physical activity is a secondary preventive goal that may be hard to achieve. In the EUROASPIRE IV survey only 40% of the participants attained a physical vigorous intensity level for at least 20 min one or more times per week [8]. In Sweden, the overall participation rate in exercise training within cardiac rehabilitation programs 1 year post-AMI is 51% [9]. Two years after AMI, 45.5% of our patients reported participating in moderate regular exercise that caused them to perspire. Thus, it appears that, as in the rest of Europe, a comparable number of our Swedish patients with coronary artery disease participate in cardiac rehabilitation. Furthermore, in our study physical exercise during leisure activity was comparable with that observed in the EUROASPIRE IV. Another example is smoking habit. Regrettably, despite that smoking cessation reduces the risk of a new myocardial infarction [16], about one third of the patients still smoked in EUROASPIRE IV survey study [8]. A similar trend was seen in the Swedish myocardial infarct registry [9] 1 year after the index AMI. Our study suggests that there is an improvement in the number of patients who quit smoking from the index AMI, since 87.5% of the study participants were classified as non-smokers 2 years after AMI. However, it is not clear whether the improved smoking cessation rate observed in our cohort could be attributed to an increase in the death rate in those patients that did continue smoking but did not survive long enough to be invited. The better results could therefore simply reflect a selection bias in our cohort because participation rates were low. Finally, guideline-directed medical therapy and glucose control 2 years after AMI were in accordance with those reported in EUROASPIRE IV [8] and SEPHIA [9], except that double anti-platelet therapy was indicated only 1 year after AMI and thus could not be adequately evaluated after 2 years.

Secondly, our study provided new information about negative developments in some risk factors from previous short-term studies. Compared with SEPHIA (9), our findings demonstrated failure of long-term secondary prevention as shown by decreased goal achievement in LDL <1.8 mmol/L from 51% in SEPHIA to 18,5% in our study, and in blood pressure control with SBP of <140 mmHg from 73% in SEPHIA to 57% in our study, notwithstanding that guideline-directed medical therapy were similar regardless of short or long term after AMI. However, caution must be taken because two studies are not fully comparable.

### Limitations

Our study has some limitations. Patients who were too sick or disabled to attend the study visits could not participate. Moreover, patients who did not speak or understand Swedish were excluded. It is possible that this category of patients may show less compliance and thus if they had been included, the goal achievements might have been even lower. Furthermore, in our study, more STEMI than NTSEMI were included despite NSTEMI constitutes the majority of ACS. There are two possible explanations: 1) the majority of the participants (87%) in our study were hospitalized at Sahlgrenska University Hospital at the time of their index event, the only hospital in Gothenburg with round-the-clock service for PCI, thus more STEMI than NTSEMI were included 2) many patients with NSTEMI have more co-morbidity and thus more hospital visits. This might explain why they were less interested in participating in the study since it means additional hospitals visits. Finally, we could not interview the patients who died during the 2 years that passed between the index AMI and the study inclusion stage. These patients were probably less compliant with the secondary prevention interventions than those who were included in the study. Thus, our results, if anything, may exaggerate the number of persons who potentially achieve secondary prevention goals, further underlining the fact that much could be gained in this area with better adherence to guidelines. Finally, this study was not powered to investigate cause-effect relationship between cardiovascular events and secondary prevention.

## Conclusion

Secondary prevention 2 years after AMI proved suboptimal in our cohort of patients. Only 3.5% of our patients attained all six secondary prevention goals. Therefore, there is considerable potential to raise the standard of preventive cardiology care through more effective lifestyle intervention and to more rigorously control of risk factors. Perhaps most importantly we need to increase awareness of the current situation and improve regular follow up. Such an effort should help to reduce cardiovascular morbidity and mortality in this patient group.

## Abbreviations

ACE: Angiotensin-converting enzyme
AMI: Acute myocardial infarction
ARB: Angiotensin II receptor blockers
BMI: Body mass index
CABG: Coronary artery bypass grafting
DBP: Diastolic blood pressure
HDL: High-density lipoprotein
ICD-10: The International Classification of Diseases
LDL: Low-density lipoprotein
PCI: Percutaneous coronary intervention
SBP: Systolic blood pressure
STEMI: ST-segment elevation myocardial infarction
TIA: Transient ischemic attack
